# Screening for chronic kidney disease: change of perspective and novel developments

**DOI:** 10.1097/MNH.0000000000001016

**Published:** 2024-08-13

**Authors:** Dominique van Mil, Lyanne M. Kieneker, Hiddo J.L. Heerspink, Ron T. Gansevoort

**Affiliations:** a Department of Nephrology; b Department of Clinical Pharmacy and Pharmacology, University Medical Center Groningen, Groningen, The Netherlands

**Keywords:** albuminuria, chronic kidney disease, cost-effectiveness, population screening, targeted screening

## Abstract

**Purpose of review:**

Chronic kidney disease (CKD) is a serious health issue because of its rising global prevalence and its complications, such as kidney failure and cardiovascular disease (CVD). CKD is mainly diagnosed late or undiagnosed, delaying or missing the initiation of preventive interventions. Screening can prevent or delay progressive kidney function decline and CVD. This article reviews diagnostic tests and risk prediction developments for patients with CKD, highlights key evidence for targeted screening, and provides new insights into population-wide screening.

**Recent findings:**

Large cohort studies and clinical trial data established the strong association of albuminuria with CKD outcomes, supporting the role of albuminuria as target of CKD screening and treatment. Significant advances in both risk prediction of CKD and CVD in CKD patients and treatment options provided new evidence for the relevance and implications of CKD screening. Guidelines recommend targeted screening in high-risk patients, but evidence suggests limited adherence to guideline recommendations. More recently, population-wide screening has been investigated as another approach, showing potential effectiveness and cost-effectiveness.

**Summary:**

There is increasing evidence for the methods, implications, and effectiveness of CKD screening. Implementing and optimizing screening strategies requires enhanced awareness and understanding of the possibilities for CKD screening within different healthcare systems.

## INTRODUCTION

The high global prevalence of chronic kidney disease (CKD) and its associated complications, such as cardiovascular disease (CVD), place a significant health and economic burden on societies [1,2,3^▪^,4^▪^]. Importantly, this burden is expected to increase as the prevalence of CKD rises because of aging populations and increasing prevalence of CKD-related risk factors, such as hypertension, obesity, and type 2 diabetes. Both lifestyle and expanding pharmacological interventions can reduce the risk of progression of CKD and the development of CKD-related complications [5^▪▪^,^6^]. CKD is mainly diagnosed late because of its asymptomatic nature, and recent studies have shown that CKD remains undiagnosed in a substantial proportion of people [2,3^▪^,^7^,^8^,9^▪^]. This delays or misses the initiation of preventive interventions. Continuous efforts are required to identify these patients in early stages, and the urgency for these efforts is increasingly recognized by important stakeholders in the field. A recent statement from the American Heart Association (AHA) proposed the concept of the cardiovascular–kidney–metabolic syndrome, underlining the interaction between kidney disease, metabolic risk factors, and CVD. Even more recently, a joint statement from the International Society of Nephrology, the European Renal Association, and the American Society of Nephrology calls for the inclusion of kidney disease in the current WHO list of major noncommunicable diseases causing premature mortality. Thus, the importance of early CKD detection is apparent. Screening for CKD plays a pivotal role in this, enabling the prevention of progressive kidney function decline and the occurrence of CVD by timely and effective implementation of kidney and cardioprotective interventions.

Discussions on CKD screening mainly focus on the methodology used for the screening, the implications, and the effectiveness of the screening. In this review article, we summarize the recent literature and developments on screening for CKD concerning these discussion points. We first discuss the most appropriate diagnostic tests to use for CKD screening and implications of CKD screening regarding risk prediction. Second, we describe key evidence for targeted CKD screening in high-risk populations. Although it is commonly assumed that CKD screening should initially be aimed at individuals with established CKD-related risk factors, population-wide screening is another option. Therefore, in the last part of the review, we provide new insights for population-wide CKD screening. 

**Box 1:**
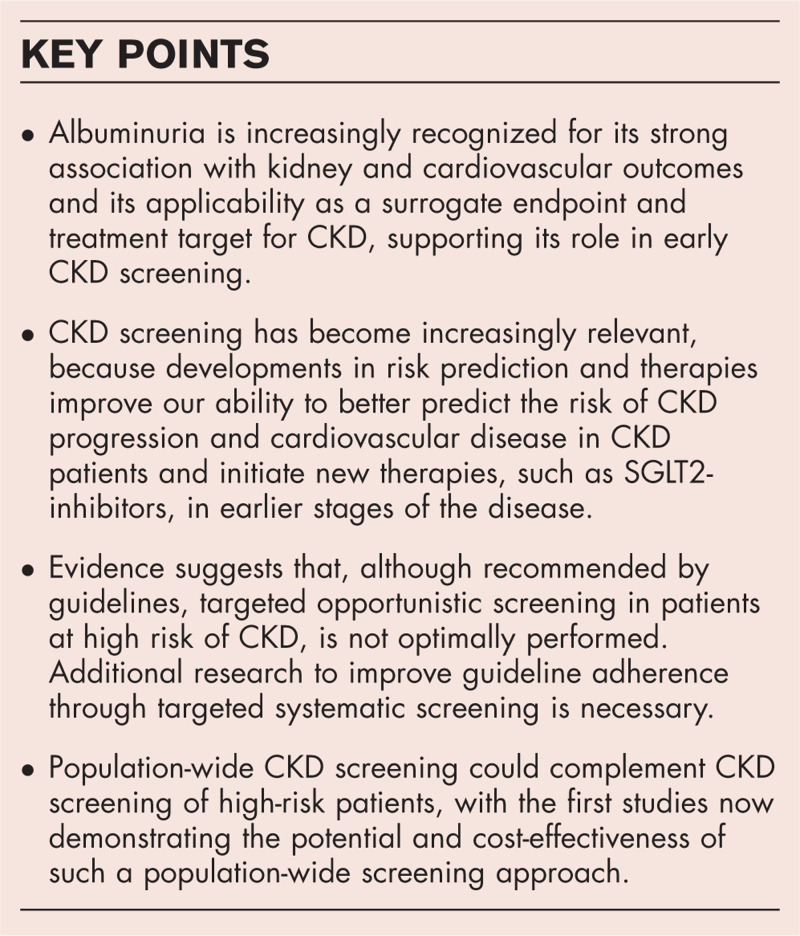
no caption available

## DIAGNOSTIC TESTS FOR CHRONIC KIDNEY DISEASE SCREENING

Various measurement methods are available to detect CKD early. A scoping review by Okpechi *et al.*[10] from 2021 reported that methods for early CKD identification widely vary. This variation exists in the type of measurement (kidney function, albuminuria, proteinuria), the number of measurements (only once or more), and applied definitions of abnormalities [e.g. CKD diagnosis based only on estimated glomerular filtration rate (eGFR), severely increased albuminuria, or both]. This has implications for the reported prevalences, diagnostic yield, and therapeutic consequences of screening. Efforts should thus be undertaken to standardize methods for early detection.

Since 2012, CKD has been defined by abnormalities of the kidney structure or kidney function, present for at least 3 months with implications for health, and this definition has been widely accepted [5^▪▪^]. The consensus is, therefore, that CKD screening should consist of dual assessment, in clinical practice most often measured by the eGFR and damage to the kidney structure, characterized by increased albuminuria, that is, the urinary albumin-to-creatinine ratio (UACR) [11]. Importantly, measurements should be repeated within and beyond the 3-month period to confirm the diagnosis and chronicity.

### Estimated glomerular filtration rate versus urinary albumin-to-creatinine ratio as diagnostic test for chronic kidney disease screening

Before the KDIGO (Kidney Disease Improving Global Outcomes) guideline of 2012, eGFR was the most considered marker for CKD screening and detection. Research has demonstrated that eGFR is strongly related to kidney outcomes and cardiovascular and all-cause mortality [12,13]. Nevertheless, eGFR may be less suited for early identification of CKD, as it can be considered a late rather than an early indicator of CKD [14]. In practice, eGFR is considered an accurate, relatively inexpensive test for screening that is widely available and easy to implement [11]. However, the actual costs and availability may differ substantially between low-income, middle-income, and high-income countries. After the KDIGO guideline update in 2012, kidney damage received more attention as part of the CKD definition, and, as a result, albuminuria emerged as a screening test to identify CKD early. Compared with eGFR, albuminuria can be considered an early indicator of CKD [14]. Albuminuria levels generally increase before the kidney function detectably declines. Work from, amongst others, the CKD Prognosis Consortium (CKD-PC) and PREVEND, has repeatedly demonstrated that albuminuria is strongly associated to kidney and cardiovascular outcomes, independently of and often stronger than eGFR [12,13,15]. Another important argument for UACR as a CKD screening test is the increasing evidence that (early) change in albuminuria can serve both as a surrogate endpoint and target for intervention. Observational data and clinical trial data showed that reducing albuminuria was strongly associated with a reduction of CKD progression, as explained in more detail in a previous issue of this journal [16]. This association appears to be stronger in patients with a higher baseline ACR [17,18]. In addition, more absolute benefits and often also more relative benefits of cardioprotective and kidney protective treatments are seen in patients with higher UACR levels compared with those with lower UACR levels [19,20]. For measuring albuminuria, UACR tests, preferably from the first-morning void, are accurate and can be performed easily at low costs in most healthcare settings [11]. These arguments support the role of albuminuria in CKD screening.

## NOVEL IMPLICATIONS OF CHRONIC KIDNEY DISEASE SCREENING

The burden of CKD is well known, as briefly mentioned above, laying the foundation for the necessity of CKD screening. Recent research has added evidence to the relevance and implications of CKD screening. This concerns developments in the risk prediction of clinical outcomes in CKD patients as well as improvements in treatment options for this patient group.

### Improvement in risk prediction in chronic kidney disease patients

In recent years, many efforts have been directed toward the risk prediction of CKD progression and risk prediction of CVD in CKD patients. Table 1 provides an overview of externally validated risk prediction equations, some of which are discussed in the following. Several risk prediction tools were developed to estimate the risk of kidney failure in patients with CKD that are recommended by the recently updated KDIGO guideline to use in clinical practice [5^▪▪^]. These risk prediction tools are specifically recommended for people with CKD stage G3-G5 (eGFR <60 ml/min/1.73 m^2^). One example is the Kidney Failure Risk Equation (KFRE), which incorporates age, sex, eGFR, and ACR in the four-variable equation. It is considered highly accurate and validated in large cohorts [21,22]. Remarkably, this risk score's performance was shown not to improve when incorporating historical information on kidney function or eGFR slopes. For patients with earlier stages of the disease, the CKD-PC risk equation is an example of a validated model, published in 2022 that enables the prediction of a 40% decline in GFR [23].

We are now also able to better predict cardiovascular risk in patients with CKD (Table 1). Perhaps most applicable for clinical practice, researchers were able to modify existing CVD models to include CKD markers. In 2023, Matsushita *et al.*[24^▪▪^] published a CKD add-on package for the SCORE2 and SCORE2-OP algorithms [Systematic Coronary Risk Evaluation (older people)]. Adding eGFR and ACR to the algorithms improved the prediction of CVD risk for patients with and without CKD. Even more recently, the PREVENT (Predicting Risk of CVD Events) equations from the American Heart Association were published, predicting CVD events among US adults without known CVD [25^▪▪^]. In these equations, eGFR is included in the main model, and adding ACR as an optional predictor improved risk prediction for those individuals with albuminuria. These developments in risk prediction imply that once individuals are identified using CKD screening, clinicians are able to predict the patient's individual risk for outcomes accurately.

**Table 1:** Selection of recommended cardiovascular disease and chronic kidney disease risk prediction tools by Kidney Disease Improving Global Outcomes^a^ for chronic kidney disease populations, published after 2010

		Required variables		
Equation, year of publication	Recommended population for use	Kidney variables (eGFR/UACR)	Other variables	Prediction outcome
**Prediction of CVD risk, no CVD history**				
QRISK3, 2017 [26]	Adults aged 25–84 years Inclusion of CKD variables in model *Developed and validated in UK cohorts*	–	Twenty variables including CKD history (recorded diagnosis of CKD stage 3–5)	Risk of CVD *10-year horizon*
SCORE2 and SCORE2-older people (OP), CKD add-on, 2023 [24^▪▪^]	SCORE2: adults aged 40–69 years SCORE2-OP: adults aged ≥70 years *CKD-Add on developed and validated in 68 cohorts worldwide; SCORE2 and SCORE2-OP are intended for risk prediction in Europe*	Primary add-on: eGFR and UACR Secondary add-on: eGFR Secondary add-on: eGFR + dipstick proteinuria	Age, sex, SBP, total cholesterol, HDL cholesterol, smoking status, diabetes status	Risk of CVD and CVD mortality *10-year horizon*
PREVENT, 2024 [25^▪▪^]	Adults 30–79 without CVD at baseline Inclusion of CKD variables in model *Developed and validated in US cohorts, intended for risk prediction in US*	eGFR, UACR as optional variable	Age, sex, SBP, total cholesterol, HDL cholesterol, BMI, diabetes status, smoking status, antihypertensive and lipid-lowering medication use + HbA1c and ZIP as optional variables	Risk of CVD *10-year and 30-year horizon (30-year horizon for age 30–59 only)*
**Prediction of CKD risk**				
KFRE (Kidney Failure Risk Equation), 2011, 2023 [21,22]	CKD G3-G5 *Developed and validated in > 60 cohorts worldwide*	eGFR, UACR	Four-variable version: age, sex eight-variable version: + calcium, albumin phosphate, bicarbonate	Kidney failure *2-year and 5-year horizon*
KPNW model (Kaiser Permanente North West), 2017 [27]	CKD G3-G5 *Developed and validated in US cohorts*	eGFR, UACR	Age, sex, SBP, antihypertensive use, diabetes, and diabetes complications	Kidney failure *5-year horizon*
Z6-score, 2022 [28]	CKD G3-G5 *Developed and validated in European cohorts*	UACR	SCr, albumin, cystatin C, urea, hemoglobin	Kidney failure *5-year horizon*
Risk of eGFR decline, CKD-prognosis consortium, 2022 [23]	CKD G1-G2 *Developed and validated in 43 cohorts worldwide*	eGFR, UACR	Age, sex, SBP, BMI, antihypertensive use, history of heart failure, coronary heart disease, and atrial fibrillation, smoking status, diabetes (and if diabetes: diabetes medication and HbA1c)	40% decline in eGFR *3-year horizon*
Klinrisk, 2022 [29]	CKD G1-G2 *Developed and validated in Canadian cohorts*	eGFR, UACR	Age, sex, and 18 other laboratory analyses from complete blood count and chemistry panels	40% decline in eGFR or kidney failure *2-year and 5-year horizon*
KidneyIntelx, 2021 [30]	CKD G1-G2, with diabetic kidney disease *Developed and validated in US cohorts*	eGFR, UACR	TNFR1, TNFR2, KIM-1, and clinical variables including SBP	Rapid kidney function decline (eGFR decline of ≥5 ml/min/1.73 m^2^/year), 40% decline in eGFR or kidney failure *5-year horizon*
**Prediction of CKD and CVD risk combined**				
Advanced CKD risk tool, 2018 [31]	CKD G4+ *Developed and validated in 29 cohorts worldwide*	eGFR, UACR	Age, sex, race, SBP, history of CVD, diabetes, smoking history	Timing and occurrence of clinical outcomes (CVD, kidney failure, death) *2-year and 4-year horizon*

BP, blood pressure; CKD, chronic kidney disease; CVD, cardiovascular disease; eGFR, estimated glomerular filtration rate; HbA1c, hemoglobin a1c; KDIGO, Kidney Disease Improving Global Outcomes; KIM-1, kidney injury molecule-1; SCr, serum creatinine; TNFR2, tumor necrosis factor receptor 2; TNRF1, tumor necrosis factor receptor 1; UACR, urinary albumin-to-creatinine ratio; US, United States.

a Ref. [5^▪▪^].

### Improvement in treatment options for chronic kidney disease patients

At the same time, treatment options for patients with CKD have also improved. After the introduction of strict blood pressure control and specifically RAAS inhibitors as kidney-protective and cardioprotective drugs for CKD patients, it has taken a long time before new therapies for CKD were developed. However, novel developments enable us to improve the prognosis of CKD patients. One of the breakthroughs is the use of sodium-glucose cotransporter-2 (SGLT2) inhibitors. Large cardiovascular and kidney outcome trials have shown that SGLT2 inhibitors reduce these outcomes in patients with CKD with and without type 2 diabetes [32–34]. This breakthrough has led KDIGO to recommend treating CKD patients with type 2 diabetes and an eGFR at least 20 ml/min/1.73 m^2^ with an SGLT2 inhibitor, irrespective of the level of albuminuria. In CKD patients without type 2 diabetes, it is recommended to start an SGLT2 inhibitor in case of an eGFR at least 20 ml/min/1.73 m^2^ with a UACR at least 200 mg/g (20 mg/mmol) or heart failure, irrespective of the UACR. The use of SGLT2 inhibitors is only suggested (instead of recommended) in CKD patients with an eGFR at least 20/ml/min/1.73 m^2^ without albuminuria (UACR < 200 mg/g or <20 mg/mmol) [5^▪▪^]. Moreover, several other novel drug classes appear to add further kidney benefit and albuminuria reduction specifically for patients with CKD and type 2 diabetes, also when they are treated with RAAS or SGLT2 inhibitors, such as glucagon-like peptide-1 (GLP-1) receptor agonists and the nonsteroidal mineralocorticoid receptor antagonist (MRA) finerenone [35,36]. Other novel kidney-protective drug classes are in late-stage clinical development to improve the CKD treatment arsenal, such as endothelin receptor antagonists, soluble guanylate cyclase activators, and aldosterone synthase inhibitors [37,38]. This implies that after identifying patients through CKD screening, we have more treatment options to offer to these patients, in order to decrease the risk of CKD and CVD outcomes. It is expected that more evidence will be added to enhance the use of the widened treatment arsenal of CKD in the upcoming years.

## SCREENING: TARGETED TO HIGH-RISK POPULATIONS?

### Current guideline recommendations for chronic kidney disease screening

One approach for CKD screening would be to screen in high-risk populations. This is also regarded as targeted screening, in which screening is performed in populations with common CKD risk factors that are essentially at high risk for CKD progression. For some years, national and international guidelines have recommended CKD screening (i.e. assessment of eGFR and albuminuria) for individuals with individuals with diabetes, hypertension, and CVD, which can be considered opportunistic screening [39–41]. It is generally accepted that CKD screening in these high-risk groups is likely to be cost-effective [11]. Additionally, as concluded by the KDIGO Controversies Conference on Early Detection and Intervention in CKD in 2021, screening should be performed in individuals with other risk factors, such as older age, systemic diseases, genetic risk factors, lower socioeconomic status, and high-risk occupational and environmental exposure [11]. The KDIGO 2024 guideline has adopted this recommendation and provides a screening algorithm for the diagnosis and staging of CKD in adults at risk for CKD, giving the highest priority for CKD screening to individuals with hypertension, diabetes, and CVD [5^▪▪^]. Screening is advised to be performed annually, but this advice is an expert-based opinion. Whether annual screening is the most appropriate and cost-effective time interval needs additional study.

### Adherence to guidelines for chronic kidney disease screening

Despite clear guideline recommendations, adherence to these guidelines is far from optimal. Folkerts *et al.*[42] investigated retrospectively the adherence to CKD screening among US patients with newly diagnosed type 2 diabetes and found that almost 85% of the patients underwent eGFR testing during a 1-year follow-up, but only 43.3% underwent UACR testing during that year. The UACR testing rates, assessed in a meta-analysis by Shin *et al.*[43], including large worldwide research cohorts, among individuals with diabetes or hypertension, respectively, were also alarmingly low at 35.1 and 4.1% during a 2-year window with a large variation between included cohorts. Low screening rates were confirmed by Chu *et al.*, showing that in a large US cohort of patients with diabetes or hypertension, only 17.5% had undergone UACR testing during a 2-year window. When stratified for diabetes status, the authors found that UACR testing was lower among nondiabetic patients with hypertension (5.1%) compared with patients with diabetes (52.3%) [44]. These data indicate that guidelines are selectively adhered to, with less frequent UACR testing compared with eGFR testing, especially in hypertensive patients.

### Improving chronic kidney disease screening by targeted systematic screening programs

Several efforts have been made to evaluate targeted systematic screening programs to improve guideline adherence for targeted CKD screening. Such programs concern systematic screening interventions directly aimed at the identification of CKD in high-risk populations as opposed to opportunistic screening of high-risk individuals as recommended by the guidelines. Two reviews from 2016 and 2022 concluded that targeted systematic screening may be effective [10,45]. However, there is a wide variation in how screening is performed, with the majority of included studies reporting results based on single albuminuria or eGFR measurements. One example of a targeted systematic screening program for CKD is the See Kidney Disease (SeeKD) project from 2016, which effectively identified adults with risk factors for CKD using a questionnaire to identify individuals at risk and subsequent eGFR assessment and urinalysis of those identified as being at risk [46]. Unfortunately, albuminuria was not measured, and CKD was defined based on a single eGFR assessment only. No recent studies investigated targeted systematic screening measuring both albuminuria and eGFR. We recently completed a study in which we invited patients from general practitioners and pharmacy patients with CKD risk factors for home-based albuminuria screening. Preliminary results show a low response rate of only 22% in a pharmacy setting and a response rate of 40% in a general practitioner setting. These data question whether targeted systematic screening is a worthwhile option.

## SCREENING: POPULATION-WIDE?

### Paving the way for population-wide screening

An approach for early CKD detection to overcome the issues mentioned above would be population-wide CKD screening, which could make CKD testing more accessible to all possible patients. In addition, one might argue that high-risk screening rules out an important patient population at risk, namely those individuals who are yet to be identified as being high-risk and those with CKD without risk factors such as hypertension or diabetes. Population-screening could provide an opportunity to screen for CKD in addition to targeted opportunistic CKD screening as currently endorsed by guidelines. Such a general population-screening should be aimed at individuals of an age at which the risk for clinical outcomes is high enough to justify the start of preventive care, whilst at the same time, there is enough time for these preventive steps to be beneficial.

Going beyond the traditional view of only screening populations at risk endorsed by the guidelines mentioned above, the European Society of Cardiology (ESC) 2021 guideline on CVD prevention in Clinical Practice has taken it a step further [41]. In this guideline, it is considered to perform opportunistic CKD screening by means of the kidney function and albuminuria in all individuals in whom cardiovascular risk estimation is indicated. This includes all individuals with any known major vascular risk factor, men older than 40 years and women who are menopausal or older than 50 years in the absence of known atherosclerotic CVD risk factors. This emphasizes once more that CKD screening is highly relevant in patients without proven risk factors, and a change of perspective is perhaps necessary. At the same time as kidney health gains a more prominent role, efforts have been undertaken to pave the way for population-wide screening.

### Population-wide screening studies

In recent years, various studies and analyses have been conducted to investigate and improve the effectiveness of population-screening in various ways, which are summarized below.

**FIGURE 1:**
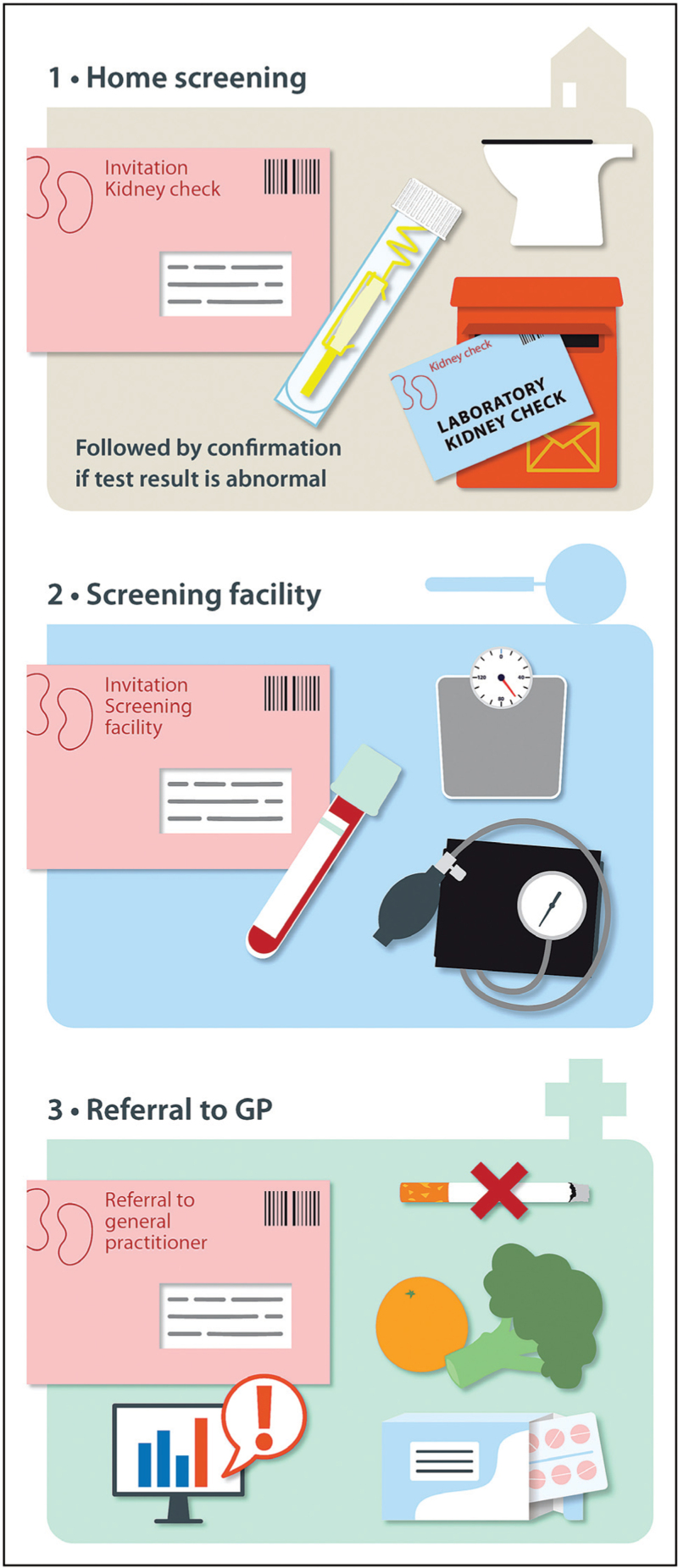
Design of a home-based strategy for albuminuria general population screening using a urine collection device, as applied in the *THOMAS* study. Data from [47^▪▪^]. (1) Individuals receive a test kit with a CE-marked PeeSpot UCD. Individuals are instructed to collect midstream urine from an early morning void using the UCD and send this UCD via mail to a central laboratory. The UCD contains a urine absorption pad that prevents bacterial growth for 4 days at room temperature, and after centrifugation, the urine is released with an albumin and creatinine recovery of 100%. Urine albumin and creatinine concentrations are measured using standard laboratory methodology. The *THOMAS* study showed a sensitivity of the UCD of 96.6% and a specificity of 97.3% [47^▪▪^]. If the first test result indicates increased albuminuria (e.g. urine albumin-to-creatinine ratio ≥3 mg/mmol), individuals are sent a second UCD for confirmation. If the second test result is negative (no albuminuria), a third confirmation test is sent. An individual is considered to have confirmed albuminuria when at least two tests indicate increased albuminuria. (2) In case of confirmed albuminuria, the individual is invited for an elaborate screening at a screening facility. During this elaborate screening, the individual is screened for CKD and CVD risk factors. (3) If individuals are identified that warrant treatment according to the prevailing guidelines, the individual is referred to their general practitioner for evaluation of the findings and prescription of treatment. CKD, chronic kidney disease; CVD, cardiovascular disease; THOMAS, Towards Home-Based Albuminuria Screening; UCD, urine collection device.

Recently, we performed a prospective trial, the Towards Home-Based Albuminuria Screening (THOMAS) study, to investigate the effectiveness of population-wide albuminuria screening for CKD, bringing along two novel techniques: home-based screening and screening via smartphone technology. The design of such a home-based screening is shown in Fig. 1. Our study randomized 15 074 Dutch individuals aged 45–80 years to home-based screening using a urine collection device (UCD) or a smartphone application [47^▪▪^]. With the UCD, urine was collected at home by the individual and sent by post to a central laboratory to assess the ACR. With the smartphone application, the ACR was measured semi-quantitatively with a dipstick at home. We found a higher participation rate for the UCD technology compared with the smartphone technology (59.4 vs. 44.3%) and showed that the UCD technology was able to identify individuals with increased albuminuria and yet unknown or sub-optimally treated CKD and cardiovascular risk factors, who would benefit from treatment optimization. Whereas the home-based screening had a high participation rate, remarkably, only half of the patients with yet unknown or sub-optimally treated risk factors visited their general practitioner. Of those, only 66% received the proposed treatment optimization, making the case for improving awareness of CKD among both patients and healthcare providers. Subsequently, we performed a cost-effectiveness analysis of THOMAS [48^▪^]. Previous studies showed limited cost-effectiveness of population screening, in contrast to targeted screening, which is considered to be cost-effective [49]. In summary, our study suggested that home-based albuminuria screening of the general population is cost-effective at an incremental cost-effectiveness ratio (ICER) of €9.225 per quality-of-life year (QALY) gained. This ICER can be deemed acceptable well below commonly applied willingness-to-pay thresholds. Incremental costs of the screening compared with usual care were €1.607 and the incremental QALY gain was 0.17 years. Moreover, the screening prevented the occurrence of kidney and cardiovascular events compared with usual care. We believe that the use of a home-based setting, the inclusion of cardiovascular outcomes, and current guideline treatments for CKD and CVD contributed to the effectiveness of our study. This is in contrast to prior cost-effectiveness studies in which population screening did not appear to be cost-effective, but differences in critical aspects between these analyses and our study explain the different results. In contrast to our analysis, screening was modelled using a general practitioner-consultation setting rather than a home-setting, and savings and gains related to cardiovascular outcomes were often not accounted for. Also, only screening for eGFR or severely increased albuminuria was considered, limiting preventive options, and these studies were performed in an era when only RAS inhibition was available.

In addition, Cusick *et al.*[50^▪^] also assessed the cost-effectiveness of population-wide screening for albuminuria among adults in the United States. The authors found one-time screening of the general population at the age of 55 years, with the addition of SGLT2-inhibition treatment on top of conventional RAAS-inhibition therapy for those identified with CKD, to be cost-effective at an ICER of $86 300 per QALY. This ICER ranged from $82 100 to $95 800 per QALY for all investigated age groups (35–75 years) and increased to $92 500 per QALY when repeating the screening every 10 years until age 75 years. Whilst no cardiovascular outcomes were considered in this study, which could further enhance the cost-effectiveness, these findings are promising and add evidence to the benefits of population-wide screening.

## CONCLUSION

Evidence gathered during the last decades supports a change of perspective in the field of CKD screening. Not only has albuminuria proven to be a suitable target for screening, enabling better identification of CKD patients, but we can now also provide these patients with better risk estimates and treatment. While guidelines have recommended targeted screening of high-risk patients for several years, guideline adherence appears to be suboptimal, further paving the way for population-wide screening. The first studies have now shown the potential and cost-effectiveness of such a population-wide screening. Implementation of screening, be it targeted or population-wide, requires a more detailed understanding of optimal screening strategies (e.g. optimal age ranges and intervals) and resource availability (e.g. in lower income countries). In the upcoming years, additional research is needed to optimize screening in high-risk populations and support the integration of population-wide CKD screening in healthcare systems globally. One example of upcoming research is a large prospective screening study from the Dutch Check@Home consortium that will investigate the optimization of home-based albuminuria screening in the general population aged 50–75 years and subsequent implementation of care after screening [51]. Such steps in the field of CKD screening are necessary in order to close the diagnostic gap in CKD care and enable the start of novel kidney and cardioprotective treatments in patients identified with CKD.

## Acknowledgements

*None.*

### Financial support and sponsorship

*R.T.G has received funding for albuminuria population screening programs from The Dutch Kidney Foundation, Top Sector Life Sciences & Health of the Dutch Ministry of Economic Affairs, and Dutch Research Council (NWO). All money was paid to the institution.*

### Conflicts of interest

*D.M. and L.M.K. report no conflicts of interest. In the past 3 years, R.T.G. has received fees for consultancy or grants, or both, for research from AbbVie, AstraZeneca, Baxter, Bayer, Healthy.io, Roche, and Sandoz. H.J.L.H. has received fees for consultancy or grants, or both, for research from AbbVie, AstraZeneca, Bayer, Boehringer Ingelheim, Chinook, CSL Behring, Dimerix, EliLilly, Gilead, Goldfinch, Merck, Novartis, NovoNordisk, Janssen, and Travere Pharmaceuticals.*

## REFERENCES AND RECOMMENDED READING

Papers of particular interest, published within the annual period of review, have been highlighted as:

▪ of special interest
▪▪ of outstanding interest
